# Transcriptome Analysis Reveals the Molecular Basis of Overfeeding-Induced Diabetes in Zebrafish

**DOI:** 10.3390/ijms241511994

**Published:** 2023-07-26

**Authors:** Guodong Ge, Jing Ren, Guili Song, Qing Li, Zongbin Cui

**Affiliations:** 1 Guangdong Provincial Key Laboratory of Microbial Culture Collection and Application, State Key Laboratory of Applied Microbiology Southern China, Institute of Microbiology, Guangdong Academy of Sciences, Guangzhou 510070, China; 2 State Key Laboratory of Freshwater Ecology and Biotechnology, Institute of Hydrobiology, Chinese Academy of Sciences, Wuhan 430072, China

**Keywords:** diabetes mellitus, zebrafish, liver, RNA-seq, signaling pathway

## Abstract

Diabetes has gradually become a serious disease that threatens human health. It can induce various complications, and the pathogenesis of diabetes is quite complex and not yet fully elucidated. The zebrafish has been widely acknowledged as a useful model for investigating the mechanisms underlying the pathogenesis and therapeutic interventions of diabetes. However, the molecular basis of zebrafish diabetes induced by overfeeding remains unknown. In this study, a zebrafish diabetes model was established by overfeeding, and the molecular basis of zebrafish diabetes induced by overfeeding was explored. Compared with the control group, the body length, body weight, and condition factor index of zebrafish increased significantly after four weeks of overfeeding. There was a significant elevation in the fasting blood glucose level, accompanied by a large number of lipid droplets accumulated within the liver. The levels of triglycerides and cholesterol in both the serum and liver exhibited a statistically significant increase. Transcriptome sequencing was employed to investigate changes in the livers of overfed zebrafish. The number of up-regulated and down-regulated differentially expressed genes (DEGs) was 1582 and 2404, respectively, in the livers of overfed zebrafish. The DEGs were subjected to KEGG and GO enrichment analyses, and the hub signaling pathways and hub DEGs were identified. The results demonstrate that sixteen genes within the signal pathway associated with fatty acid metabolism were found to be significantly up-regulated. Specifically, these genes were found to mainly participate in fatty acid transport, fatty acid oxidation, and ketogenesis. Furthermore, thirteen genes that play a crucial role in glucose metabolism, particularly in the pathways of glycolysis and glycogenesis, were significantly down-regulated in the livers of overfed zebrafish. These results indicate insulin resistance and inhibition of glucose entry into liver cells in the livers of overfed zebrafish. These findings elucidate the underlying molecular basis of zebrafish diabetes induced by overfeeding and provide a model for further investigation of the pathogenesis and therapeutics of diabetes.

## 1. Introduction

Diabetes is a major contributor to mortality around the world. The global number of diabetes cases is projected to increase from approximately 537 million today to 783 million by 2045. It is estimated that around 6.7 million adults between the ages of 20 and 79 have died as a result of diabetes or its complications. In China, the number of people with diabetes was 140.9 million in 2021 [1]. Diabetes mellitus (DM) is caused by insufficient insulin secretion or decreased sensitivity of cells to insulin, leading to high blood glucose levels for an extended period followed by damage to many tissues and organs in the body, such as kidney failure, cardiovascular disease, nerve and brain damage, and other microvascular complications [2]. There are three main types of diabetes mellitus: type 1 diabetes (T1DM), type 2 diabetes (T2DM), and gestational diabetes.

T2DM is the most common type of diabetes mellitus, accounting for 95% of diabetes cases, and begins with insulin resistance. The body obtains glucose from three main sources: the intestinal absorption of food, the breakdown of glycogen (glycogenolysis), and gluconeogenesis. Insulin is the principal hormone that regulates the uptake of glucose from the blood into most cells of the body, especially the liver, adipose tissue, and muscle. Therefore, a deficiency of insulin or the insensitivity of its receptors plays a central role in all forms of diabetes mellitus [3]. Insulin not only promotes the absorption of glucose but also stimulates the synthesis of fat. High triglyceride levels and hepatic steatosis are associated with insulin resistance [4,5].

Many people with T2DM have evidence of prediabetes such as impaired fasting glucose and/or impaired glucose tolerance before meeting the criteria for T2DM [6]. The impaired glucose tolerance in particular is a major diagnosis risk factor for progression to full-blown diabetes mellitus. High levels of cholesterol and triglycerides in the blood, as well as increased systolic and diastolic blood pressure, are also risk factors for developing diabetes [7]. Patients with diabetes often experience metabolic syndrome such as dyslipidemia characterized by abnormal cholesterol and triglyceride metabolism [8,9,10]. Cardiovascular disease (CVD) is the primary cause of morbidity and mortality in T2DM patients. One significant factor that increases the risk of CVD in diabetic patients is atherosclerotic lipid abnormalities, and the increased levels of cholesterol and triglycerides in the blood are important markers for lipid evaluation [11]. The previous studies indicate that hepatic lipid metabolism is also significantly disturbed in individuals with T2DM [12,13].

T2DM is primarily caused by lifestyle factors and genetics. A number of lifestyle factors are known to be important in the development of T2DM, including obesity (defined by a body mass index greater than 30), lack of physical activity, poor diet, stress, and urbanization [14,15]. Dietary factors, such as sugar-sweetened drinks, are associated with an increased risk [16,17,18]. The type of fats in the diet are also important factors since saturated fat and trans fats increase the risk and polyunsaturated and monounsaturated fat decrease the risk [19]. Excessive consumption of white rice may increase the risk of diabetes, especially in Chinese and Japanese people [20]. The progression of prediabetes to overt T2DM can be slowed or reversed by lifestyle changes or medications that improve insulin sensitivity or reduce the liver’s glucose production [21].

However, molecular mechanisms underlying the development of diabetes remain largely not elucidated. Zebrafish have been widely used in the field of metabolic disease research. In terms of diabetes research, zebrafish possess several characteristics. Zebrafish have a similar blood glucose regulation mechanism to mammals [22], and several approved T2DM drugs can significantly lower blood glucose in zebrafish high models [23]. Additionally, zebrafish have been used to study the mechanisms of pancreatic β-cell regeneration and to screen compounds that promote pancreatic β-cell regeneration [24]. In terms of fat metabolism, zebrafish have been used to study the fat metabolism and the development of weight-loss drugs [25], and to screen and evaluate drugs that can lower cholesterol levels in the body [26]. However, zebrafish have a relatively low blood volume, which is not conducive to performing large-scale and repetitive blood sampling experiments [27], such as studying the mechanisms of blood glucose metabolism regulation. Considering the conservation of lipid metabolism across different species, the zebrafish is considered a good model for studying metabolic disorders such as diabetes and obesity [28], and the use of the zebrafish as a model organism to study diabetes has gained recognition over time. Multiple techniques have been developed to establish zebrafish diabetes models, including chemical methods, dietary induction, glucose soaking, and gene knockout [29]. The zebrafish model of diabetes is employed for investigating stem cell therapy for treating diabetes [30] and diabetic heart failure [31]. Dietary-induced diabetes leads to increased insulin secretion in the pancreases of zebrafish [23,32]. Nevertheless, the alterations at the molecular level within zebrafish diabetes induced by dietary factors remain undisclosed.

In this study, we generated a zebrafish model of diabetes by overfeeding. We found that overfed zebrafish had excessive accumulation of lipid droplets in their liver. Comparative analysis by high-throughput RNA-seq revealed novel molecular factors that contribute to lipid droplet accumulation and insulin resistance in overfed zebrafish. These findings support the successful induction of zebrafish diabetes by overfeeding and provide a model for further studying the etiology and treatment of diabetes.

## 2. Results

### 2.1. The Elevation of Fasting Blood Glucose Levels in Male Zebrafish after Four Weeks of Overfeeding

**Figure 1:**
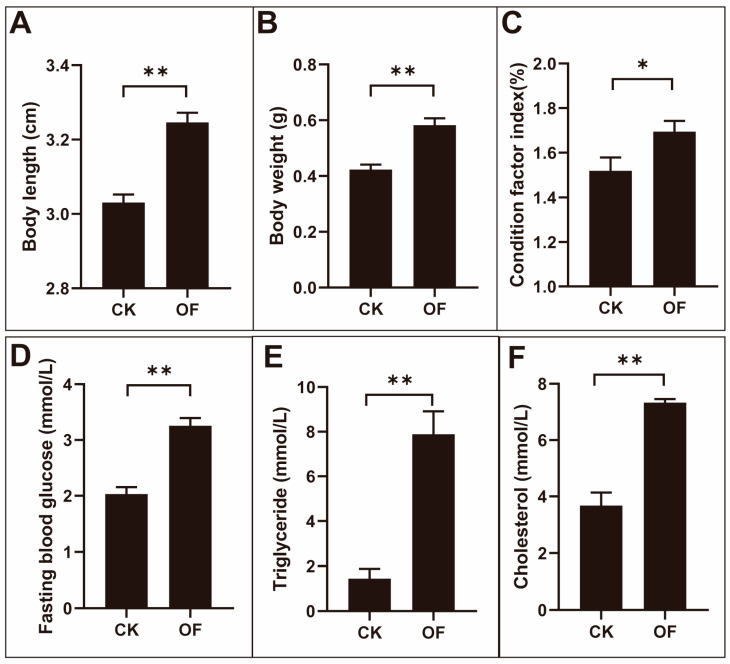
The variation of body index, fasting blood glucose, triglycerides, and total cholesterol in male zebrafish after overfeeding. The body length (**A**), body weight (**B**), condition factor index (**C**), fasting blood glucose (**D**), and the levels of triglycerides (**E**) and total cholesterol (**F**) in the serum of male zebrafish were significantly increased in the overfed groups (*, *p* < 0.05; **, *p* < 0.01; n = 13).

The body length, body weight, and condition factor index of male zebrafish in the overfed (OF) group were significantly higher than those in the control (CK) group after overfeeding for four weeks. Among them, the average body length of zebrafish in the CK group was 3.03 cm, while the average body length of zebrafish in the OF group was 3.25 cm. The average weight of zebrafish in the CK group was 0.42 g, whereas the average weight of zebrafish in the OF group was 0.58 g. The average condition factor index of zebrafish in the CK group was 1.52, and the average condition factor index of zebrafish in the OF group was 1.69. After one month of overfeeding, the body length, body weight, and condition factor index of zebrafish increased 1.1-fold, 1.4-fold, and 1.1-fold, respectively, when compared to those of the CK group (Figure 1A–C).

In addition, the average level of fasting blood glucose in OF group was 3.25 mmol/L, which was significantly higher than that of the CK group at 2.03 mmol/L (Figure 1D and Figure S1A). However, the fasting blood glucose level of female zebrafish in the OF group did not show a significant change compared to that in the CK group (Figure S1B).

The average level of triglycerides in the serum of the CK group and OF group was 1.44 mmol/L and 7.89 mmol/L, respectively. The level of triglycerides in the serum of the OF group was increased 5.5-fold when compared to that in the CK group (Figure 1E). The average level of cholesterol in the serum of the CK group and OF group was 3.67 mmol/L and 7.33 mmol/L, respectively. The level of cholesterol in the serum of the OF group was significantly increased twofold compared to that in the CK group (Figure 1F).

Together, these data indicate that the OF male zebrafish have developed characteristics of diabetes after four weeks of overfeeding.

### 2.2. The Hepatic Accumulation of Lipid Droplets in Male Zebrafish after Four Weeks of Overfeeding

**Figure 2:**
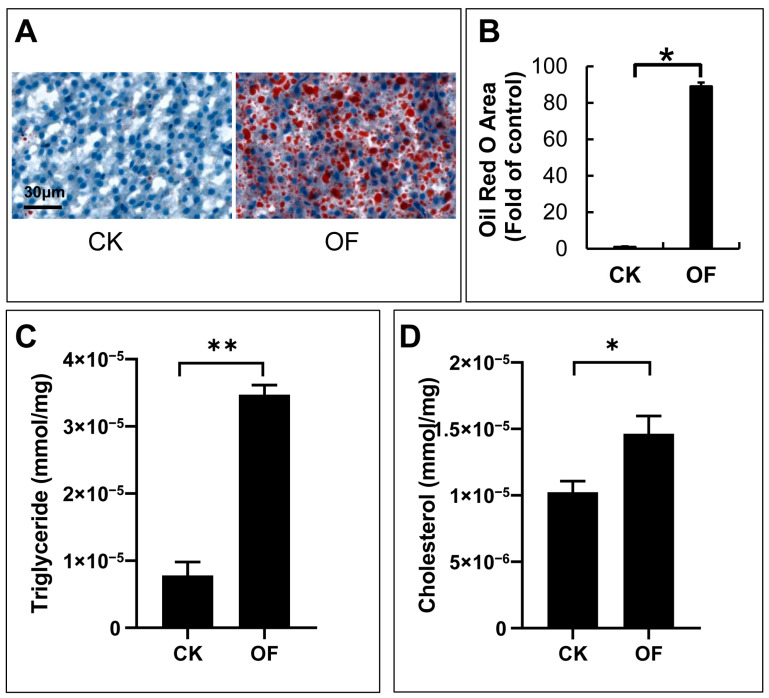
Accumulation of lipid droplets in livers of overfed zebrafish. Images of Oil Red O staining for section of livers in control and overfed groups (**A**). The fold change of lipid droplet accumulation according to the results of Oil Red O staining between the control group and the overfed group were quantified by Image J software (version 1.50i) and showed in histogram (**B**). The levels of triglyceride (**C**) and cholesterol (**D**) from liver in control and overfed group. (Values are means ± SEM; *, *p* < 0.05; **, *p* < 0.01; CK: control group, OF: overfed group; n = 5.)

The liver plays an important role in regulating the level of blood sugar. Sections of the liver in the CK and OF groups were stained with oil red O. An increased accumulation of lipid droplets in the livers of OF zebrafish was found (Figure 2A). When quantified by ImageJ, the area of the oil red region increased 80-fold in the OF group when compared with that in the CK group (Figure 2B).

The levels of triglycerides and cholesterol in the livers of zebrafish were also significantly affected by overfeeding. The average level of triglycerides in the livers of the CK and OF groups was 0.78 × 10^−5^ mmol/mg and 3.47 × 10^−5^ mmol/mg, respectively. Compared with that of the control group, the level of triglycerides in the livers of the OF zebrafish increased 4.5-fold (Figure 2C).

The average levels of cholesterol in the livers of the CK and OF groups were 1.02 × 10^−5^ mmol/mg and 1.46 × 10^−5^ mmol/mg, respectively. Compared with that of the CK group, the level of cholesterol in the livers of the OF zebrafish increased 1.4-fold (Figure 2D).

These findings suggest that overfeeding has a severe effect on lipid metabolism in zebrafish, leading to the accumulation of lipids in the liver.

### 2.3. RNA-Seq Analysis to Identify Differentially Expressed Genes

To identify the phenomenon of insulin resistance at the molecular level in the livers of the OF group, transcriptome analysis of the livers in the CK and OF groups was performed (Figure S2A). The raw data of RNA-sequencing ranged from 19.29 to 22.81 M. After removing the adapters and low-quality data, the clean reads ranged from 19.25 to 22.77 M, and the ratio of clean reads was above 99% in all groups, indicating that the quality of RNA-sequencing data is reliable (Figure S2B). The clean reads in each group were mapped to the reference genome of zebrafish, and the mapping rate was above 92% (Figure S2B).

**Figure 3:**
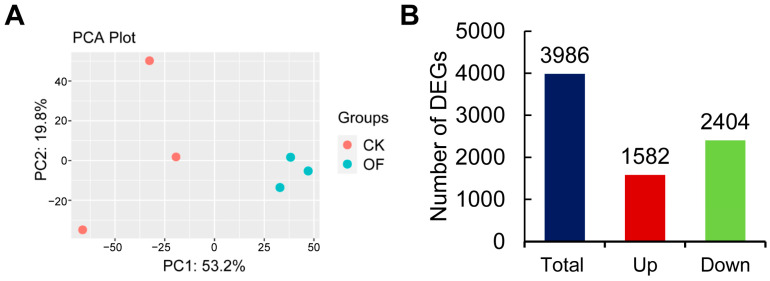
The results of data analysis for the transcriptome of livers in control and overfed groups. Principal component analysis (PCA) of gene expression profiles for differentially expressed genes (**A**). The number of differentially expressed genes (DEGs) for up- and down-regulated in zebrafish liver after overfeeding is shown in the histogram (**B**). (Fold change ≥ 1.5 and *p*-value ≤ 0.05.)

The principal component analysis (PCA) showed that the OF group and the CK group were clustered together, respectively, and the differences between the groups were obviously identified (Figure 3A). A total of 3986 differentially expressed genes (DEGs) were obtained, including 1582 up-regulated and 2404 down-regulated DEGs (fold change ≥ 1.5 and *p*-value ≤ 0.05) (Figure 3B and Table S1).

### 2.4. KEGG Enrichment Analysis for DEGs

**Figure 4:**
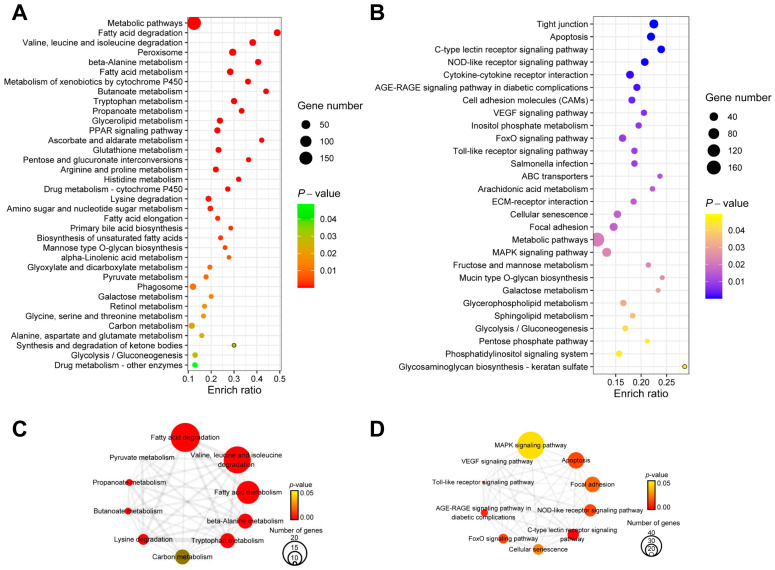
The results of KEGG enrichment analysis for DEGs in livers after overfeeding. Dot plot of KEGG enrichment analysis results for up-regulated DEGs (**A**) and down-regulated DEGs (**B**). Network of the top 10 hub pathways with the highest maximal clique centrality (MCC) for up-regulated DEGs (**C**) and down-regulated DEGs (**D**). (Edges between nodes represent Jaccard similarity coefficients; the colors and sizes of nodes stand for the *p*-value of pathways and the number of genes in the pathway, respectively.)

KEGG enrichment analysis of DEGs was performed using the online software KOBAS (version 3.0) (Table S2). The most enriched KEGG pathways (*p*-value ≤ 0.05) of up-regulated DEGs were metabolic pathways, fatty acid degradation, valine, leucine and isoleucine degradation, peroxisome, and beta-alanine metabolism. The most enriched KEGG pathways (*p*-value ≤ 0.05) of down-regulated DEGs were tight junction, apoptosis, the C-type lectin receptor signaling pathway, the NOD-like receptor signaling pathway, and the cytokine–cytokine receptor interaction (Figure 4A,B).

Since different KEGG signaling pathways may share the same DEGs, the Jaccard similarity coefficient was introduced to calculate the distance between two signaling pathways based on the proportion of shared DEGs (Table S3). The networks of KEGG pathways for up- and down-regulated DEGs were obtained. Then, the hub pathways in the networks were identified using CytoHubba. Among the signaling pathways enriched from up-regulated DEGs, the top five hub pathways were metabolic pathways, fatty acid degradation, valine, leucine and isoleucine degradation, tryptophan metabolism, and lysine degradation (Figure 4C and Table 1). Among the signaling pathways enriched from down-regulated DEGs, the top five hub signaling pathways were the C-type lectin receptor signaling pathway, the AGE-RAGE signaling pathway in diabetic complications, the NOD-like receptor signaling pathway, apoptosis, and the toll-like receptor signaling pathway (Figure 4D and Table 2). Additionally, twenty-two DEGs were mapped to the KEGG pathway of fatty acid degradation, which was the first of the hub signaling pathways enriched from up-regulated DEGs (Figure 4C and Table S2). Among the hub signaling pathways enriched from down-regulated DEGs, forty-seven DEGs were mapped to the MAPK signaling pathway, which was the first of hub pathways (Figure 4D and Table S2).

**Figure 5:**
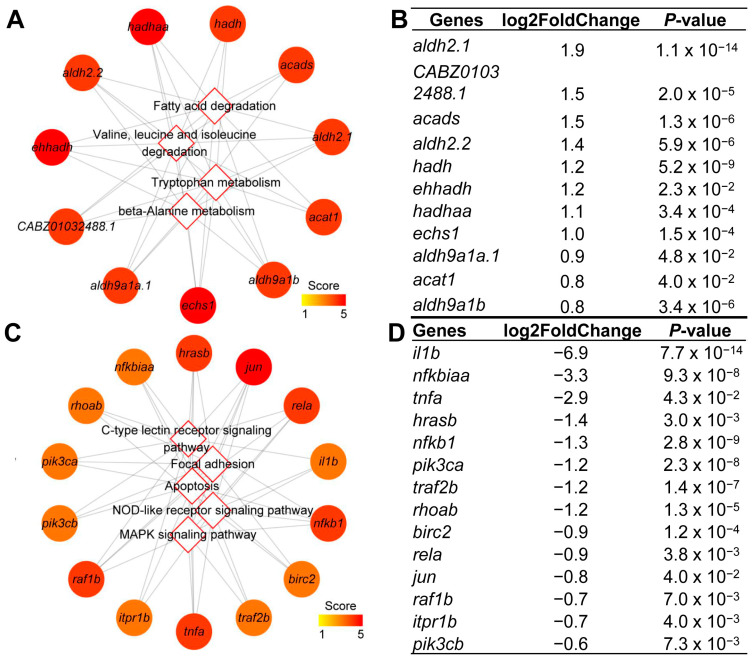
Identified hub genes in the hub pathways. Network of the 11 hub genes mapped to top 4 hub pathways enriched from up-regulated DEGs (**A**). The fold change of up-regulated hub genes after overfeeding (**B**). Network of 14 hub genes mapped to top 5 hub pathways enriched from down-regulated DEGs (**C**). The fold change of down-regulated hub genes after overfeeding (**D**). Round and diamond stand for genes and pathways, respectively; edge represents the gene mapped to the pathway in (**A**,**C**).

A total of eleven up-regulated hub DEGs were shared by the hub pathways of valine, leucine and isoleucine degradation, fatty acid degradation, tryptophan metabolism, and lysine degradation (Figure 5A). Among these hub DEGs, eight were up-regulated at least one-fold, including *aldh2.1*, *CABZ01032488.1*, *acads*, *aldh2.2*, *hadh*, *ehhadh*, and *ehhadh* (Figure 5B). Additionally, a total of fourteen hub down-regulated DEGs were shared by the hub pathways of C-type lectin receptor signaling pathway, AGE-RAGE signaling pathway in diabetic complications, NOD-like receptor signaling pathway, apoptosis, and toll-like receptor signaling pathway (Figure 5C). Among these hub DEGs, eight were down-regulated at least one-fold, including *il1b*, *nfkbiaa*, *tnfa*, *hrasb*, *nfkb1*, *oik3ca*, *traf2b*, and *rhoab* (Figure 5D).

### 2.5. GO Enrichment Analysis of DEGs

GO enrichment analysis of DEGs was performed using KOBAS. A total of 133 GO terms were enriched from up-regulated DEGs, including 58 terms related to biological processes, 15 GO terms related to cellular component, and 60 GO terms related to molecular function (Table S4). Additionally, a total of 238 GO terms were enriched from down-regulated DEGs, including 117 GO terms related to biological processes, 30 GO terms related to cellular component, and 91 GO terms related to molecular function (Table S4).

**Figure 6:**
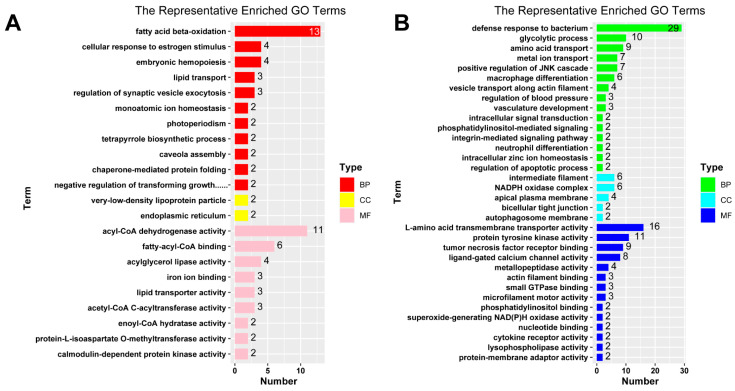
Bar plots for representative terms of GO enrichment analysis. The GO terms for up-regulated DEGs (**A**) and down-regulated DEGs (**B**) were reduced using the REVIGO tool and shown in bar plots. The different aspects of representative GO terms are shown in different colors. BP: biological process; MF: molecular function; CC: cellular component.

The online software REVIGO (version 1.8.1) was used to perform redundancy analysis to obtain the representative GO terms. In biological processes, the representative GO terms enriched from up-regulated DEGs include fatty acid beta-oxidation (GO:0006635), cellular response to estrogen stimulus (GO:0071391), embryonic hemopoiesis (GO:0035162), lipid transport (GO:0006869), and regulation of synaptic vesicle exocytosis (GO:2000300). In molecular function, the representative GO term enriched from up-regulated DEGs include acyl-CoA dehydrogenase activity (GO:0003995), fatty-acyl-CoA binding (GO:0000062), acylglycerol lipase activity (GO:0047372), iron ion binding (GO:0005506), lipid transporter activity (GO:0005319), and acetyl-CoA C-acyltransferase activity (GO:0003988) (Figure 6A).

The representative biological processes enriched from down-regulated DEGs include defense response to bacterium (GO:0042742), glycolytic process (GO:0006096), amino acid transport (GO:0006865), metal ion transport (GO:0030001), and positive regulation of JNK cascade (GO:0046330). The representative cellular component includes intermediate filament (GO:0005882), NADPH oxidase complex (GO:0043020), apical plasma membrane (GO:0016324), bicellular tight junction (GO:0005923), and autophagosome membrane (GO:0000421). The representative molecular function includes L-amino acid transmembrane transporter activity (GO:0015179), protein tyrosine kinase activity (GO:0004713), tumor necrosis factor receptor binding (GO:0005164), ligand-gated calcium channel activity (GO:0099604), and metallopeptidase activity (GO:0008237) (Figure 6B).

### 2.6. Overfeeding Up-Regulated Fatty Acid Metabolism Genes and Down-Regulated Glucose Metabolism Genes in Zebrafish Liver

**Figure 7:**
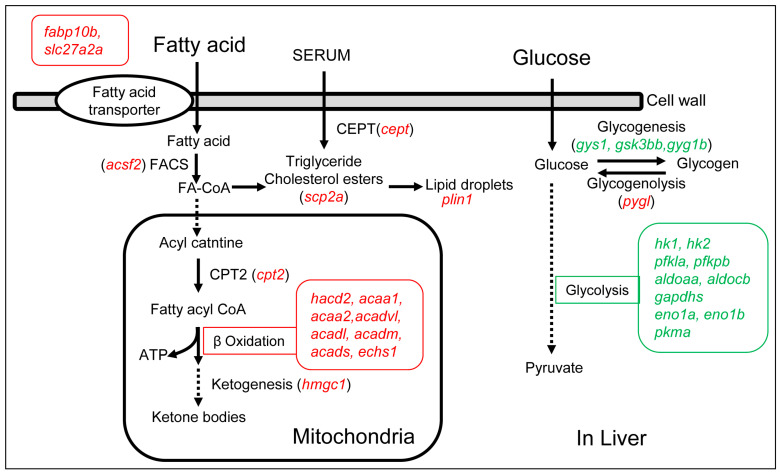
The diagram of the up- and down-regulated genes in the signaling pathways of fatty acid metabolism and glucose metabolism in the livers of the overfed group. The letters in italic and red represent up-regulated genes, the letters in italic and green represent down-regulated genes.

Based on the analysis of DEGs in the livers of OF zebrafish, the main signaling pathways affected by overfeeding were fatty acid and glucose metabolisms (Figure 7).

Sixteen DEGs from the up-regulated DEGs were found in the signaling pathway of fatty acid metabolism. Among them, *plin1* was up-regulated 14-fold, which encodes a protein that is a lipid droplet-associated protein that mainly attaches to the surface of lipid droplets. The other 15 DEGs mainly function in processes such as fatty acid transmembrane transport (*fabp10b*, *slc27a2a*), fatty acid beta-oxidation (*hacd2*, *acaa1*, *acaa2*, *acadl*, *acadm*, *acads*, *acadvl*, *echs1*), ketone body production (*hmgcl*), cholesterol transfer (*scp2a*, *cetp*), carnitine palmitoyltransferase (*cpt2*), and acetyl-CoA synthase (*acsf2*) (Figure 7 and Figure S3, and Table 3).

Furthermore, 13 DEGs from the down-regulated DEGs were mapped to the signaling pathway of glucose metabolism. Two DEGs with the most down-regulated fold change were *hk2* (down-regulated by 6-fold) and *gyg1b* (down-regulated eightfold). These down-regulated DEGs mainly function in glycolysis (*hk1*, *hk2*, *pfkla*, *pfkpb*, *aldoaa*, *aldocb*, *gapdhs*, *eno1a*, *eno1b*, *pkma*) and glycogen generation (*gys1*, *gsk3bb*, *gyg1b*). Additionally, one DEG (*pygl*) involved in glycogen decomposition was up-regulated 2.5-fold (Figure 7, Figure S4 and Table 4). These results suggest that the glucose content decreases in the liver cells of OF zebrafish and that glucose uptake is blocked.

Overall, the activity of fatty acid metabolism is enhanced while the activity of glucose metabolism is inhibited in the livers of OF zebrafish, indicating that the source of energy supply has shifted from glucose metabolism to fatty acid metabolism in the livers of OF zebrafish, which is consistent with the physiological phenomenon of T2DM.

## 3. Discussion

The main characteristic of T2DM is insulin resistance and compensatory inadequate insulin secretion, resulting in elevated blood glucose levels. Insulin resistance refers to the decreased response of the liver, muscles, and adipose tissue to insulin, leading to symptoms such as hyperglycemia, dyslipidemia, visceral obesity, and elevated inflammatory factors [33]. Insulin levels in the blood are increased during the early stages of T2DM; however, long-term glucose stimulation can cause toxicity to the pancreatic β-cell, leading to ER stress and cell apoptosis in the later stages of diabetes, which results in a decline in the ability of the β-cell to synthesize and secrete insulin, thus leading to a decrease in insulin levels in the blood plasma [34]. Diabetics are often associated with elevated serum cholesterol and triglycerides [7]. Thus, insulin resistance can be evaluated by measuring triglyceride levels, cholesterol content, and glucose tolerance in the serum [35]. In this study, after one month of overfeeding, the body length, body weight, condition factor index, fasting blood glucose level, and serum triglycerides and cholesterol contents of male zebrafish were significantly higher than those in the control group, indicating that we have successfully established a T2DM zebrafish model by overfeeding.

The establishment of a T2DM model is mainly achieved by breaking down the sensitivity of tissues towards insulin, resulting in impaired glucose absorption and consequent elevation of blood glucose levels. Adult zebrafish were alternately immersed in water or 2% glucose solutions for 28–30 days, inducing increased fasting blood glucose levels, retinal damage, and impaired bone cell function [36,37,38]. Larvae zebrafish from 3 hpf (hours post-fertilization) to 5 dpf (days post-fertilization) that were alternately immersed in 4% and 5% glucose solutions can induce diabetic-like retinopathy [39]. Zebrafish immersed in a gradually increasing glucose solution—with 50 mM glucose solution immersion for 4 days, 100 mM glucose solution immersion for 3 days, and immersion in 200 mM glucose solution for 13 days—have exhibited symptoms of increased body weight and elevated blood glucose levels [40]. After immersing adult zebrafish in a 111 mM glucose solution for 14 days, the blood glucose level increased, the amount of glycated protein in the eyes also increased, but the transcription level of insulin receptors in the muscles decreased and the response to exogenous insulin was impaired [41].

Overweight or obese is also an important factor in the development of T2DM [42,43]; thus, an alternative method to establish a diabetes model in zebrafish is performed through overfeeding or high-fat feeding. Adult zebrafish that were overfed a commercially available fish food can exhibit symptoms of diabetes such as decreased glucose tolerance and reduced insulin expression [23,32]. Feeding zebrafish with 10% cholesterol and immersing them in a 2% glucose solution simultaneously for 19 days resulted in more symptoms of diabetes in zebrafish larvae, such as significant increases in insulin, glucagon, glucose, triglyceride, and cholesterol levels [44,45]. In obese individuals, adipose tissue releases more non-esterified fatty acids, glycerol, hormones, pro-inflammatory cytokines, and other factors. These can induce insulin resistance, accelerate pancreatic damage, and ultimately lead to T2DM [46]. In addition, insulin resistance in adipose tissue can lead to mitochondrial dysfunction, resulting in the production of ROS and inflammatory cytokines, as well as the release of adipokines, cytokines, chemokines, excessive lipids, and toxic lipid metabolites into the bloodstream. These substances further exacerbate insulin resistance in other tissues [47,48]. Moreover, insulin resistance in liver tissue leads to an increase in the accumulation of fatty acids in the liver [49]. In a previous study, the gene expression profiling of liver–pancreas in the overfed zebrafish was analyzed, and the pathways common to human T2DM were revealed [23]. In this study, we have found that the elevation of fasting blood glucose level was accompanied by a large number of lipid droplets accumulating within the livers of overfed male zebrafish. Mouse fed with high-fat diet show impaired glucose tolerance, insulin resistance, increased body weight, increased levels of triglycerides in plasma and liver, and hepatic steatosis [50]. Most of these characteristics were observed in male zebrafish after overfeeding for four weeks in this study. However, the underlying molecular basis of diabetes induced by overfeeding remains largely unknown. Therefore, we further performed transcriptome analyses to investigate novel changes in the livers of overfed zebrafish.

After four weeks of overfeeding, male zebrafish showed a significant increase in fasting blood glucose levels, while female zebrafish did not exhibit significant changes in fasting blood glucose levels even after being overfed for a duration of eight weeks. Discrepancy studies conducted worldwide have also found that there is sexual dimorphism in the incidence of diabetes, with a higher incidence rate among males than females [51]. It can be speculated that the reason for this may be excessive estrogen leading to a greater likelihood of insulin resistance in females [52].

Further studies are needed to uncover the mechanisms by which estrogen and testosterone contribute to the sexual dimorphism in diabetes between males and females.

Excess fatty acids in the liver need to be either consumed through β-oxidation or converted into cholesterol or triglycerides, and then transported to other parts of the body via apolipoprotein [53]. Excess triglycerides and cholesterol in the liver are packaged in lipid droplets [54]. Perilipin 1, expressed by *plin1*, is a lipid droplet-associated protein that primarily attaches to the surface of lipid droplets. Its functions include increasing the size of lipid droplets and regulating triglyceride levels [55]. In this study, we found the expression of this gene was up-regulated 14-fold in the livers of the overfed zebrafish.

In addition, we found that multiple other genes in the entire metabolism from fatty acid transport to fatty acid β oxidation are significantly up-regulated. For instance, the genes *slc27a2a* and *fabp10b* were up-regulated by 2.9-fold and 2.8-fold, respectively. The gene *slc27a2a* encodes a member of the long-chain fatty acyl-CoA synthetase family and is involved in lipid biosynthesis and fatty acid degradation [56]. The *fabp10b* encodes a fatty acid binding protein (FABP), which primarily promotes the uptake, intracellular transport, and metabolism of fatty acids [57]. It transports fatty acids from the cell membrane to their metabolic sites for β-oxidation and synthesis of triglycerides and phospholipids. Thus, the up-regulated of the *slc27a2a* and *fabp10b* can facilitate the transport of fatty acids from outside the cell to the inside in overfed male zebrafish. Moreover, sterol carrier protein 2a (*scp2a*) mainly regulates the transport of intracellular lipids, promoting the synthesis of triglycerides and cholesterol from exogenous fatty acids. The main function of cholesteryl ester transfer protein (*cetp*) is to transport cholesteryl esters and triglycerides, regulating the reverse transport of cholesterol and transferring excess cholesterol in peripheral tissues to the liver for metabolism and digestion. In this study, we found the expression of these two genes in the livers of overfed zebrafish were up-regulated 2.3-fold and 2.1-fold, respectively.

Among the KEGG enrichment analysis results for up-regulated DEGs, there were five signal pathways related to fatty acid metabolism. The most important hub pathways in the KEGG enrichment results were fatty acid degradation and fatty acid metabolism. The hub genes that mapped to fatty acid degradation and fatty acid metabolism were *hadh*, *ehhadh*, *acat1*, *hadhaa*, *acads*, and *echs1.* Among them, the most up-regulated gene is *acads*, which encodes Acyl CoA dehydrogenase and participates in the first step of fatty acid beta-oxidation. These hub genes can be used as potential molecular markers for diagnosis of diabetes at the early stage. In the GO enrichment analysis for up-regulated DEGs, the representative GO term was fatty acid β-oxidation, which includes 13 raw GO terms. Further analysis revealed that these up-regulated expression genes were mainly involved in fatty acid transport, fatty acid oxidation, and cholesterol transport.

The expression levels of *acsf2* and *cpt2* were up-regulated 2.5-fold and 2.7-fold, respectively, in the livers of overfed zebrafish. Long-chain fatty acids first need to be catalyzed into acyl-CoA by acyl-CoA synthetase family member 2 (*acsf2*), and then transferred into the mitochondrial inner membrane by carnitine palmitoyltransferase 2 (*cpt2*). After multiple enzyme-catalyzed reactions, the long-chain fatty acids are converted to acetyl-CoA, which can enter the tricarboxylic acid cycle for oxidation. Additionally, the genes of multiple enzymes involved in fatty acid β-oxidation were significantly up-regulated in the livers of overfed zebrafish, including Acyl CoA dehydrogenase (*acadvl*, *acadl*, *acadm*, *acads*), Enoyl CoA hydratase (*echs1*), 3-Hydroxyacyl CoA dehydrogenase (*hacd2*), and β-ketothiolase (*acaa1*, *acaa2*). The product of the β-oxidation of fatty acids, acetyl CoA, can further transform into ketone bodies and be transported to other tissues to provide energy for the body [58]. HMG-CoA lyase, encoded by the *hmgcl*, plays an important role in ketogenesis and the gene was up-regulated 1.6-fold in the livers of overfed diabetic zebrafish. Fatty acid β-oxidation is primarily regulated by the PPARα signaling pathway [59]. We found that PPARα pathway has been significantly enriched from up-regulated genes, and 15 up-regulated DEGs in the livers of overfed zebrafish were mapped to this signaling pathway. These data above indicate that overfeeding can enhance the fatty acid metabolism of the zebrafish liver. The up-regulation of these genes related to fatty acid metabolism results in an increased metabolic burden on the mitochondria, leading to the production of ROS, and mitochondrial dysfunction. Mitochondrial dysfunction and oxidative stress are largely involved in T2DM [60,61].

The metabolism of glucose in cells is primarily carried out through glycolysis [62]. However, the expression levels of the genes encoding the enzymes involved in glycolysis were significantly down-regulated in the livers of overfed zebrafish. These enzymes mainly include hexokinase (*hk1*, *hk2*), phosphofructokinase (*pfkla*, *pfkpb*), fructose bisphosphate aldolase (*aldoaa*, *aldocb*), glyceraldehyde phosphate dehydrogenase (*gapdhs*), *enolase* (*eno1a*, *eno1b*), and pyruvate kinase (*pkma*). The genes g*yg1b*, *gsk3bb*, and *gys1*, which encode protein associated with glycogen synthesis, are significantly down-regulated in the livers of overfed zebrafish. The expression of *gyg1b*, which codes for a glycogen protein with glucosyltransferase activity, is decreased sixfold, and its catalytic product is the substrate of glycogen synthase. The gene *gys1* encodes glycogen synthase, which is down-regulated 1.6-fold in the livers of overfed zebrafish. Glycogen synthase kinase (*gsk3bb*), which activates glycogen synthase, is down-regulated 2.6-fold. However, the gene *pygl*, which catalyzes glycogen breakdown, is up-regulated 2.5-fold in the livers of overfed zebrafish, indicating a decrease in glucose content and impaired glucose uptake in liver cells due to overfeeding.

In this study, four insulin receptor subunits (*irs1*, *irs2a*, *irs2b*, *irs4a*) and two insulin receptors (*insra*, *insrb*) were identified in the zebrafish liver through transcriptome. Among them, *irs1* and *insra* were up-regulated, while *irs2a*, *irs2b*, *irs4a*, and *insrb* were down-regulated. However, the fold changes of these six genes were all less than 1.5 and statistically not significant (*p*-value > 0.05) (Table S5). It is reported that the decreased phosphorylation of insulin receptors was important for the blockade of insulin signaling pathways in insulin-resistant cells [63]. Mice with heterozygous loss of the insulin receptor had normal glucose and insulin tolerance [64]. These studies suggest that the phosphorylation of insulin receptors plays a more important role in insulin signal transduction than altered expression levels of insulin receptors. In addition, numerous studies over the past years have linked the formation of lipid droplets and increased contents of cholesterol and triglyceride to insulin resistance in the liver [65,66,67,68]. In this study, an excessive accumulation of lipid droplets and increased levels of triglycerides and cholesterol occurred in the livers of overfed zebrafish, indicating the development of insulin resistance in a certain extent.

In summary, we uncovered changes in signaling molecules related to fatty acid and glucose metabolism in the livers of overfed zebrafish by using high-throughput transcriptome sequencing and bioinformatic analysis techniques. Overfed male zebrafish exhibited enhanced fatty acid metabolism and suppressed glucose metabolism, suggesting the development of insulin resistance and diabetes.

## 4. Materials and Methods

### 4.1. Methods of Overfeeding Experiments

The AB strain zebrafish used in this study were maintained under standard laboratory conditions at 28 °C with a light/dark cycle of 12/12 h.

The method of overfeeding protocol performed in this study was based on a published work [23]. Four-month-old wild type male zebrafish were selected according to their weight and body length and randomly divided into the overfed group and the control group, with 20 zebrafish in each group. The control group was fed once a day (9:00 a.m.) under a regular condition and the overfed group was fed four times a day (9:00 a.m., 11:00 a.m., 15:00 p.m., and 17:00 p.m.). Feed 4.08 g/20 tails each time.

The crude fat, crude protein, crude ash, and crude fiber contents in the main components of frozen red worms were 4.07%, 52.73%, 19.2%, and 9.56%, respectively. The calorie content is 3.2254 kcal/g.

### 4.2. Blood Collection and Measurement

The method for blood collection from zebrafish was the same as the protocol in the previous article [69]. The adult zebrafish were fasted for 18 h before blood collection. The level of blood glucose was measured using a glucose meter, Accu-Chek Performa, according to the manufacturer’s instructions.

### 4.3. Staining with Oil Red O and Histology

The liver tissues from the control and overfed groups were fixed in 4% paraformaldehyde (PFA, Beyotime Biotechnology, Shanghai, China) at 4 °C overnight. After fixation, the samples were stained with Oil Red O as previously described [70]. Photographs of the tissue sections were taken using an Aperio VERSA Brightfield, Fluorescence & FISH Digital Pathology Scanner from Leica (Wetzlar, Germany). Quantitative analysis was performed using ImageJ software.

### 4.4. Measurement of Total Cholesterol and Triglyceride

The levels of total cholesterol and triglycerides (TG) were determined with commercial kits according to the manufacturer’s instructions. The total cholesterol assay kit (A111-1-1) and triglyceride assay kit (F001-1-1) were purchased from Nanjing Jiancheng Bioengineering Institute.

### 4.5. Sample Collection and Analysis for RNA-Sequencing

Zebrafish were fasted overnight and then sacrificed after being overfed for four weeks. The livers of three zebrafish as one sample were collected and subjected to total RNA extraction using TRIzol Reagent (Invitrogen, Waltham, MA, USA), and each group contains three independent samples. The methods for sample quality analysis, preparation of the RNA library, and RNA-seq were as previously described [70]. Six sequencing libraries were then constructed and sequenced. Library construction and high-throughput RNA-sequencing were performed by experts at the Analytical and Testing Center at the Institute of Hydrobiology, Chinese Academy of Sciences (http://www.ihb.ac.cn/fxcszx/, accessed on 29 November 2022).

### 4.6. Bioinformatics Analysis

The bioinformatics analysis was conducted as previously described [71]. Briefly, the raw data were first filtered by Trimmomatic (version 0.38) to remove joints and low-quality data, and clean reads were obtained. These high-quality clean reads were then mapped to the reference genome (Danio rerio GRCz11) obtained from the NCBI assembly database using HISAT2 (version 2.1.0) [72] to obtain the BAM formation of the aligned files. Then, the counts of reads were summarized using the read summarization program featureCounts [73]. These counts were used for gene differential expression analysis using the Bioconductor DESeq2 package [74]. Low abundance genes (number of summed reads < 10) were filtered before differential expression analysis. Genes with a fold change ≥ 1.5 and a *p*-value ≤ 0.05 were considered to be differentially expressed genes (DEGs).

KOBAS-i was performed for the Kyoto Encyclopedia of Genes and Genomes (KEGG) and Gene Ontology (GO) enrichment analysis of DEGs [75]. The dot plots for KEGG enrichment results and bar plots for GO enrichment results (*p*-value ≤ 0.05) were generated by ggplot2 in R-studio. The RStudio (Version 1.4.1717) was used to calculate the Jaccard coefficients between two KEGG signaling pathways based on the number of shared genes from the enrichment analysis results, and the network diagram were created by Cytoscape (version: 3.8.2) software. The Cytoscape plug-in cytoHubba [76] was used to analyze hub signaling pathways and genes by the MCC (maximal clique centrality) method and exported the visualization. The REVIGO tool was used to cluster and prune GO terms based on the *p*-value obtained from KOBAS-i [77].

### 4.7. Statistical Analysis

Statistical analysis was performed using Microsoft Excel software for Windows (Microsoft Office 2013, Microsoft, Redmond, WA, USA). The data in this study were analyzed statistically using the independent samples *t*-test. The data are presented as mean ± standard deviation.

## 5. Conclusions

In this study, a zebrafish diabetes model was established by overfeeding. Compared with the control group, the body length, body weight, and condition factor index of zebrafish increased significantly after four weeks of overfeeding. The fasting blood glucose level increased significantly, and a large number of lipid droplets accumulated in the liver. The triglyceride and cholesterol contents in serum and liver also increased significantly. Through transcriptome sequencing of the livers of overfed zebrafish, 16 up-regulated DEGs were found to function in the signaling pathway of fatty acid metabolism, including the fatty acid transport, fatty acid oxidation, and ketogenesis. In addition, 13 down-regulated DEGs were involved in glycolysis and glycogenesis of glucose metabolism signaling pathway, indicating insulin resistance and inhibition of glucose entry into liver cells of overfed male zebrafish. These findings clarified the molecular basis of overfed -induced zebrafish diabetes and provided a foundation for further study of the pathogenesis and treatment of diabetes in a zebrafish model.

## Figures and Tables

**Table 1:** Top 10 hub terms of up-regulated genes ranked by the MCC method.

Rank	Signaling Pathway	Score
1	Metabolic pathways	2.11 × 10^8^
2	Valine, leucine and isoleucine degradation	2.11 × 10^8^
2	Fatty acid degradation	2.11 × 10^8^
4	Tryptophan metabolism	2.07 × 10^8^
5	Lysine degradation	2.07 × 10^8^
6	Carbon metabolism	1.71 × 10^8^
7	Propanoate metabolism	1.67 × 10^8^
8	Fatty acid metabolism	1.67 × 10^8^
9	Butanoate metabolism	1.63 × 10^8^
10	Beta-Alanine metabolism	1.31 × 10^8^

**Table 2:** Top 10 hub terms of down-regulated genes ranked by the MCC method.

Rank	Signaling Pathway	Score
1	C-type lectin receptor signaling pathway	8.06 × 10^7^
2	AGE-RAGE signaling pathway in diabetic complications	8.06 × 10^7^
3	NOD-like receptor signaling pathway	8.06 × 10^7^
3	Apoptosis	8.06 × 10^7^
3	Toll-like receptor signaling pathway	8.06 × 10^7^
3	FoxO signaling pathway	8.06 × 10^7^
7	Focal adhesion	8.06 × 10^7^
8	Cellular senescence	8.06 × 10^7^
9	Metabolic pathways	7.98 × 10^7^
10	VEGF signaling pathway	7.98 × 10^7^

**Table 3:** The genes associated with fatty acid metabolism in liver.

**Gene Name**	**Fold**	**Gene Description**	**Function**
*fabp10b*	2.8	fatty acid binding protein 10b, liver basic	Fatty acid transporter
*scp2a*	2.3	sterol carrier protein 2a	Cholesterol transport
*slc27a2a*	2.9	solute carrier family 27 member 2a	Fatty acid transfer
*acsf2*	2.7	acyl-CoA synthetase family member 2	Fatty acyl-CoA synthase
*cpt2*	2.5	carnitine palmitoyltransferase 2	Carnitine palmitoyltransferase
*hacd2*	2.0	3-hydroxyacyl-CoA dehydratase 2	Fatty acid β-oxidation
*acaa1*	2.3	acetyl-CoA acyltransferase 1	Fatty acid β-oxidation
*acaa2*	2.3	acetyl-CoA acyltransferase 2	Fatty acid β-oxidation
*acadl*	2.4	acyl-CoA dehydrogenase long chain	Fatty acid β-oxidation
*acadm*	2.6	acyl-CoA dehydrogenase medium chain	Fatty acid β-oxidation
*acads*	2.8	acyl-CoA dehydrogenase short chain	Fatty acid β-oxidation
*acadvl*	1.9	acyl-CoA dehydrogenase very long chain	Fatty acid β-oxidation
*echs1*	2.0	enoyl CoA hydratase, short chain, 1, mitochondrial	Fatty acid β-oxidation
*hmgcl*	1.6	3-hydroxy-3-methylglutaryl-CoA lyase	Ketogenesis
*cetp*	2.1	cholesteryl ester transfer protein, plasma	Cholesteryl ester transfer
*plin1*	14.1	perilipin 1	Lipid droplet-associated protein

**Table 4:** The genes associated with glucose metabolism in liver.

Gene Name	Fold	Gene Description	Function
*hk1*	1.9	hexokinase 1	Glycolysis
*hk2*	6.0	hexokinase 2	Glycolysis
*pfkla*	2.6	phosphofructokinase, liver a	Glycolysis
*pfkpb*	2.2	phosphofructokinase, platelet b	Glycolysis
*aldoaa*	2.0	aldolase a, fructose-bisphosphate, a	Glycolysis
*aldocb*	3.1	aldolase C, fructose-bisphosphate, b	Glycolysis
*gapdhs*	2.5	glyceraldehyde-3-phosphate dehydrogenase, spermatogenic	Glycolysis
*eno1a*	4.3	enolase 1a, (alpha)	Glycolysis
*eno1b*	2.3	enolase 1b, (alpha)	Glycolysis
*pkma*	3.4	pyruvate kinase M1/2a	Glycolysis
*gys1*	1.6	glycogen synthase 1 (muscle)	Glycogenesis
*gsk3bb*	2.6	glycogen synthase kinase 3 beta, genome duplicate b	Glycogenesis
*gyg1b*	8.1	glycogenin 1b	Glycogenesis
*pygl*	(2.5)	phosphorylase, glycogen, liver	Glycogenolysis

## Data Availability

The data presented in this study are available in the article and supplementary material.

## Supplementary Materials

The following supporting information can be downloaded at: https://www.mdpi.com/article/10.3390/ijms241511994/s1.
