# Supplementary material for: Transcriptome Analysis Reveals the Molecular Basis of Overfeeding-Induced Diabetes in Zebrafish
Source: Int J Mol Sci. 2023 Jul 26;24(15):11994. doi: 10.3390/ijms241511994 (PMC10418320; doi:10.3390/ijms241511994)
Supplement: Supplementary file 1 [file ijms-24-11994-s001.zip › SUPPLEMENTAL FIGURES AND LEGENDS.pdf]

SUPPLEMENTAL FIGURES AND LEGENDS

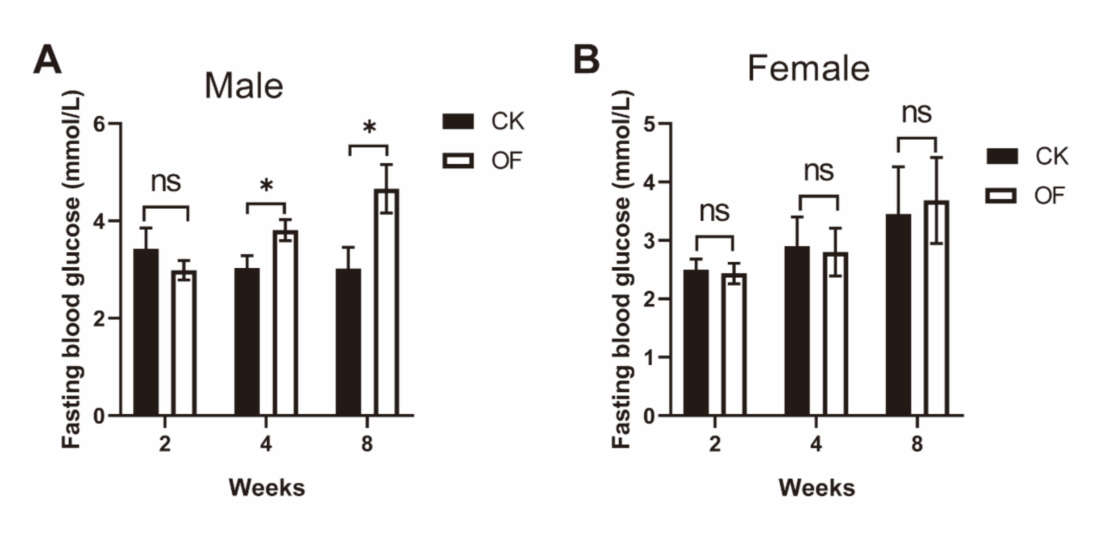

Figure S1. The differences of fasting blood glucose levels between male (**A**) and female (**B**) zebrafish were measured at two, four, and eight weeks after overfeeding. *, $p < 0.05$; ns: No Significantly; CK: control group, OF: overfed group.

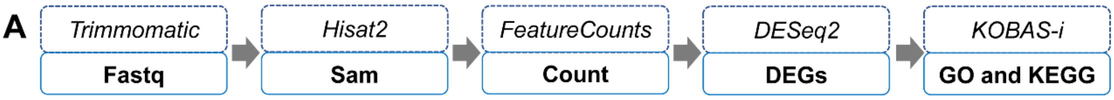

B

Samples	Raw reads(M)	Clean reads(M)	Clean reads rate	Paired reads	Paired reads rate	Unpaired reads	Unpaired reads rate	Mapping rate
	22.51	22.47	99.85%	15.88	70.66%	6.59	29.34%	92.92%
CK	20.87	20.83	99.80%	14.35	68.88%	6.48	31.12%	92.46%
	22.81	22.77	99.84%	15.94	69.99%	6.83	30.01%	92.63%
	22.15	22.11	99.81%	14.94	67.55%	7.17	32.45%	92.59%
OF	19.29	19.25	99.81%	13.13	68.22%	6.12	31.78%	92.01%
	20.90	20.86	99.81%	14.58	69.89%	6.28	30.11%	92.15%

Figure S2. The landscapes of data analysis for the transcriptome of livers in control and overfed groups. The tools for transcriptome data analysis are

shown in italics, and the format of the analysis results exported is in boldface inside the parentheses **(A).** Statistics of raw data and mapping rate of RNA-seq **(B)**.

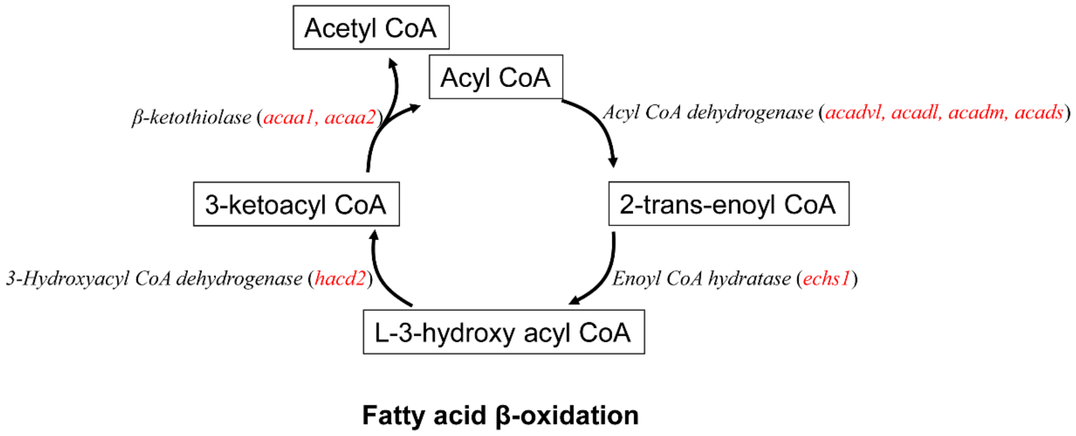

Figure S3. Up-regulated DEGs in each step of fatty acid β-oxidation were showed in red.

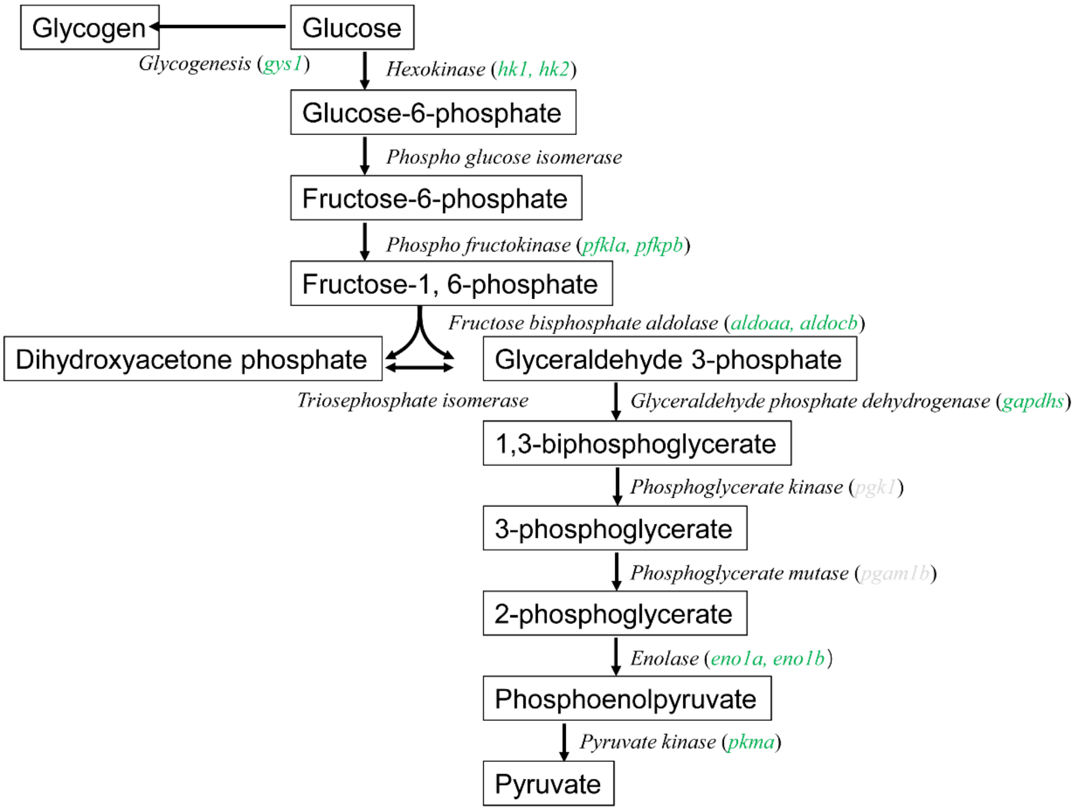

Figure S4. Down-regulated DEGs in each step of glucose metabolism were showed in green.
